# Rapid and Direct Detection of Methamphetamine in Biofluids using a MXene‐Enabled Electrochemical Sensor

**DOI:** 10.1002/advs.202521857

**Published:** 2026-01-08

**Authors:** Ri Wang, Xiaofei Deng, Bo Chen, Jienan Shen, Wei Xu, Yingjie Zhu, Yi Zhang, Hui Yang

**Affiliations:** ^1^ Institute of Biomedical and Health Engineering Shenzhen Institutes of Advanced Technology Chinese Academy of Sciences Shenzhen China; ^2^ Shenzhen Key Laboratory of Drug Addiction, the Brain Cognition and Brain Disease Institute Shenzhen Institutes of Advanced Technology Chinese Academy of Sciences Shenzhen China; ^3^ Faculty of Life and Health Sciences Shenzhen University of Advanced Technology Shenzhen China; ^4^ Shenzhen Neher Neural Plasticity Laboratory Shenzhen‐Hong Kong Institute of Brain Science Shenzhen Institutes of Advanced Technology Chinese Academy of Sciences Shenzhen China; ^5^ School of Biomedical Engineering Shenzhen University of Advanced Technology Shenzhen China

**Keywords:** drug screening, electrochemical sensors, methamphetamine, MXene, rapid detection

## Abstract

Illicit drug abuse presents a global challenge to public health and security, creating an urgent need for reliable detection methods to curb trafficking and mitigate societal harm. Methamphetamine (METH), a highly addictive and widely circulated stimulant, requires urgent monitoring solutions. We introduce an approach for the rapid and direct detection of METH using an electrochemical sensor enhanced with MXene nano‐interfaces that operates on the principle of electrochemical oxidation. Theoretical simulations are conducted to elucidate the reaction pathway of METH and to analyze the molecular interactions between METH and the MXene surface, providing key insights into the underlying molecular dynamics. The rich surface functionalities of MXene enable a favorable structural affinity with METH, facilitating enhanced interfacial interactions that promote oxidation kinetics and amplify electrochemical responses. The sensor demonstrates a linear response to METH across a concentration range from 2 ngmL^−1^ to 50 µg mL^−1^, alongside satisfactory anti‐interference performance and repeatability, rendering it appropriate for on‐site preliminary screening of positive samples. Its successful validation in complex biological matrices confirms its broad applicability. By combining user‐friendly operation with a rapid response, this method establishes a robust theoretical framework and a practical solution for the on‐site screening of METH and related illicit substances.

## Graphical abstract

A MXene‐enhanced electrochemical sensor enables the rapid and direct detection of methamphetamine. Molecular  simulations reveal that specific MXene surface functional groups act as key signal amplifiers by facilitating interfacial interactions. The sensor demonstrates high sensitivity and robust performance in complex biological matrices. This method meets on‐site preliminary screening standards, offering a promising solution for rapid field deployment.

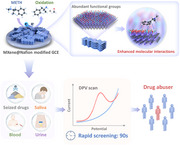

## 1 Introduction

The abuse of illicit drugs remains a global concern due to its profound threats to public health, social stability, and environmental sustainability [1, 2, 3]. According to the United Nations Office on Drugs and Crime (UNODC), worldwide drug consumption continues to rise. Among these substances, amphetamine‐type stimulants (ATS) rank as the third most abused class of illicit drugs globally, following cannabis and opioids [4]. Methamphetamine (METH), a prevalent synthetic drug, dominates the ATS market and draws significant concern from medical and judicial authorities because of its severe societal impact [5, 6]. METH is extremely addictive and neurotoxic; even small amounts exert potent stimulatory effects on the central nervous system, causing irreversible damage to the human body. These effects include heightened risks of mental disorders, neurological diseases, cardiovascular events, and mortality [7, 8, 9]. Consequently, the reliable detection and monitoring of METH are of great significance for multiple sectors, including law enforcement agencies, anti‐narcotics units, community health services, and forensic laboratories [10, 11, 12]. To curb its pervasive abuse and spread, the development of rapid, accurate, and field‐deployable screening methods is critical for maintaining social stability, safeguarding human health, and supporting evidence‐based drug prevention programs worldwide.

Traditional methods for METH detection, such as gas chromatography‐mass spectrometry (GC‐MS), liquid chromatography‐mass spectrometry (LC‐MS), high‐performance liquid chromatography (HPLC), and nuclear magnetic resonance (NMR) spectroscopy, offer high sensitivity and precise quantification [13, 14, 15]. However, these techniques rely on sophisticated laboratory equipment and involve complex sample pretreatment procedures. This results in high operational costs, prolonged analysis times, and stringent expertise requirements, making them unsuitable for on‐site rapid detection. In response, recent studies based on alternative principles have demonstrated significant potential for field‐deployable METH detection. These emerging approaches include colorimetric, fluorescent, chemiluminescent, surface plasmon resonance, surface‐enhanced Raman scattering, and electrochemical techniques [16, 17, 18, 19].

Electrochemical sensors have garnered widespread application in biosensing and chemical detection [20, 21] owing to their rapid response kinetics, low reagent consumption, compatibility with miniaturized devices, and inherent capabilities for real‐time monitoring [22, 23, 24, 25]. These collective advantages firmly position them as promising tools for the detection of METH. Initial work by Garrido et al. investigated the electrochemical oxidation properties of four amphetamine‐like drugs on a bare glassy carbon electrode (GCE), establishing that METH could be oxidized under alkaline conditions [26]. Subsequent studies further characterized the electrochemical profile of METH on alternative electrode substrates, including screen‐printed carbon electrodes (SPCE) [27] and boron‐doped diamond electrodes (BDD) [28], ultimately realizing the detection of METH in biological samples. Although these direct detection sensors offer rapid response and are suitable for on‐site analysis, their wider application is limited by insufficient sensitivity and analytical accuracy.

The ongoing development of nanotechnology and the emergence of novel nanomaterials have unlocked significant potential for enhancing the performance of direct electrochemical sensors for METH detection. A diverse array of nanostructured materials, such as graphene derivatives, carbon nanotubes, metallic nanoparticles, molecularly imprinted polymers, and metal–organic frameworks, has been successfully incorporated into METH sensing platforms, each contributing to markedly enhanced analytical performance [29, 30, 31, 32].

Among these advanced materials, MXene, a class of 2D transition metal carbides/nitrides first synthesized in 2011, has rapidly gained prominence due to its exceptional suite of properties [33]. MXene demonstrates great potential across a broad spectrum of fields, including catalysis, energy storage, electromagnetic interference shielding, sensing, and biomedicine [34, 35, 36, 37]. Among the diverse MXene structures, Ti_3_C_2_T_x_ (where T_x_ denotes surface terminations such as ─O, ─OH, or ─F) represents the most extensively studied variant. While MXene‐modified electrochemical sensors have already demonstrated enhanced performance for detecting various biological and chemical analytes [38, 39], their specific function in promoting the electrochemical oxidation of METH for direct detection had not been explored prior to this study. The fundamental electrochemical behavior of METH at MXene interfaces and the associated signal amplification mechanism remained unexplored.

**FIGURE 1:**
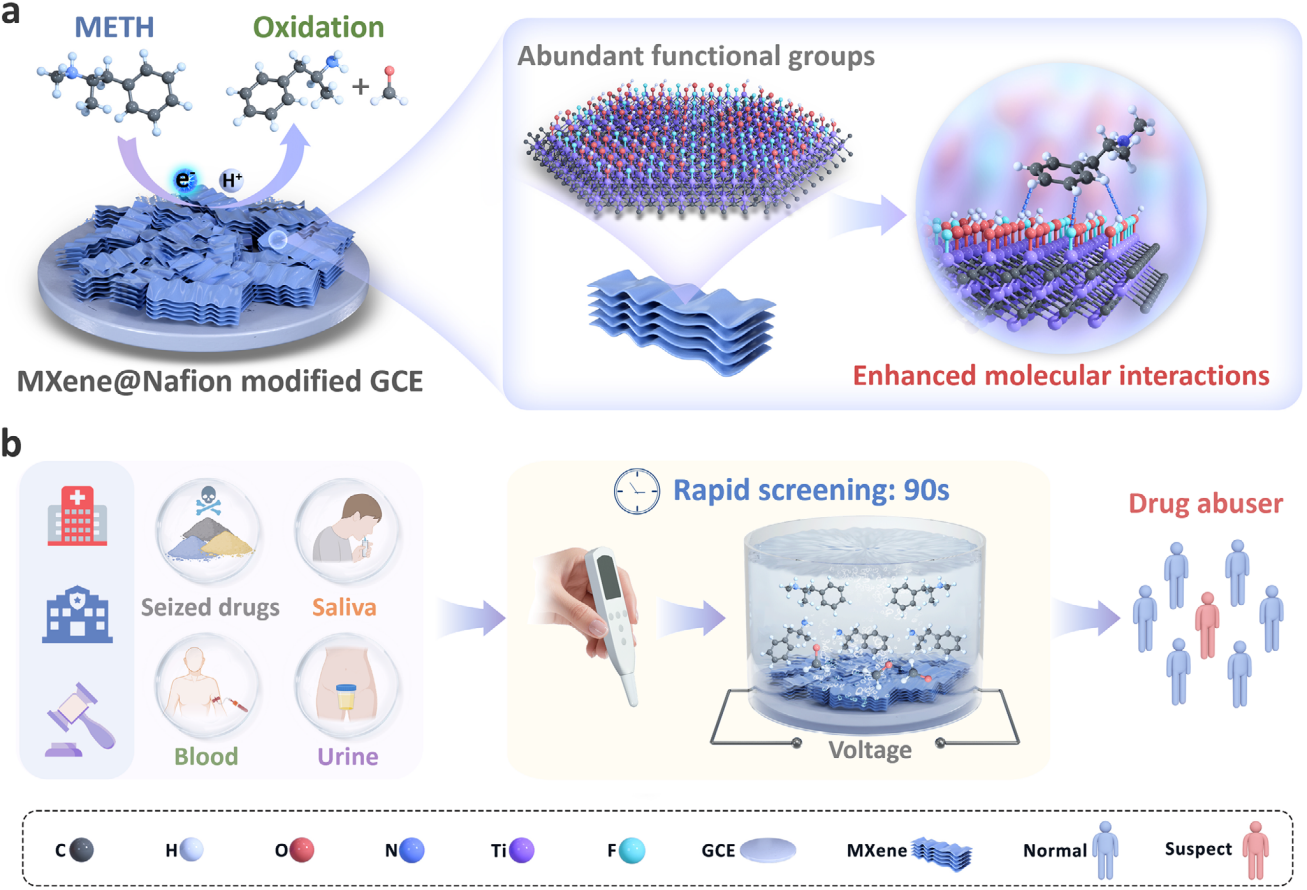
Schematic illustration of the MXene@Nafion modified electrochemical sensor for METH detection. (a) Modification of the GCE surface with multilayer MXene@Nafion nanocomposites enhances the electrochemical signal generated by METH oxidation. The rich surface functionalities of MXene play a key role in signal amplification. (b) Application of the sensor for rapid detection of METH in various biological matrices, demonstrating its utility for on‐site drug screening.

In this study, we develop a fast and sensitive electrochemical sensor modified with MXene (Ti_3_C_2_T_2_, T═O, OH, and F) and investigate the electrochemical behavior of METH on this platform based on the principle of electrochemical oxidation. As illustrated in Figure 1, a MXene@Nafion (MX@Nf) nanofilm was modified onto a GCE surface to generate and enhance the electrochemical signal. The detection mechanism was systematically explored from both simulation and experimental perspectives to analyze the electrochemical behavior of METH at the sensing interface. Molecular simulations were employed to elucidate the theoretical interactions between MXene and METH molecules, while electrochemical experiments validated the sensor's detection capabilities. Moving beyond conventional validation using spiked biological fluids, this study further assessed practical applicability by directly detecting METH in serum samples collected from rats administered with the drug. The proposed sensor, operating on electrochemical oxidation, exhibits simple operation and rapid response. Furthermore, its design facilitates seamless integration with backend detection modules, enabling the development of compact, portable handheld devices. This portability renders the system well‐suited for diverse on‐site testing scenarios in public security, clinical, and community screening settings. Its ability to analyze multiple sample types highlights its significant potential for advancing portable analytical technologies aimed at public health and forensic applications.

## 2 Results

### 2.1 Characterization of the Nanomaterials

**FIGURE 2:**
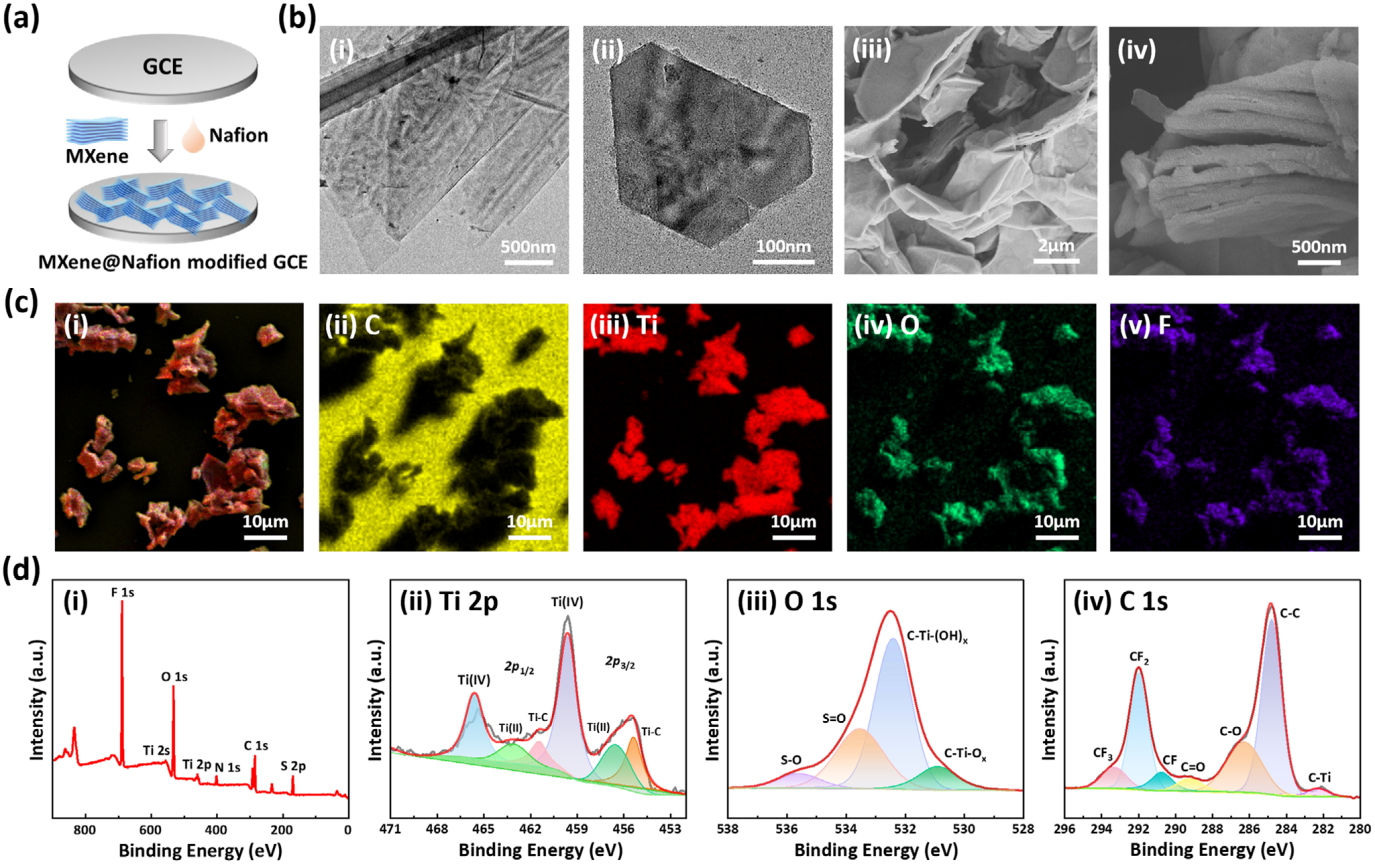
Characterization of the nanomaterials. (a) Schematic of the electrode modification process. (b) (i, ii) TEM images of the MX@Nf nanocomposites and (iii, iv) SEM images of the modified GCE surface. (c) (i) SEM image with (ii–v) associated EDS elemental maps showing the distribution of C, Ti, O, and F. (d) XPS analysis: (i) full survey scan, and high‐resolution core‐level spectra of (ii) Ti 2p, (iii) O 1s, and (iv) C 1s.

The morphological and structural properties of the nanomaterials were thoroughly characterized to understand the foundation of the sensor's performance. As illustrated in Figure 2b, transmission electron microscopy (TEM) images confirmed that the initial clay‐like MXene powder was successfully exfoliated into ultrathin nanosheets through ultrasonic processing. This transformation is critical for enhancing charge transfer rates. Subsequent scanning electron microscopy (SEM) analysis revealed that the MX@Nf composites maintained a well‐defined, layered architecture, which increases the sensor's effective surface area and provides abundant active sites to facilitate electrochemical interactions. Meanwhile, the incorporated Nafion primarily functions as a binding agent, markedly improving the adhesion and mechanical stability of the nanocomposites on the underlying electrode surface. This stable integration prevents layer detachment during measurement and ensures reliable sensor operation. These combined properties confer both high reactivity and robust durability to the MX@Nf‐modified sensor.

The distribution of the material was further validated by energy dispersive spectroscopy (EDS) elemental mapping (Figure 2c), which showed an in‐plane distribution of the primary constituent elements (C, Ti, O, and F) across the MX@Nf‐modified electrode surface. The surface chemical composition and bonding configurations of the modified electrode were analyzed by X‐ray photoelectron spectroscopy (XPS). The full survey spectra and deconvoluted high‐resolution scans are presented in Figure 2d and Figure S1, with corresponding quantitative fitting parameters provided in Table S1. All binding energies were calibrated against the adventitious carbon C 1s peak centered at 284.80 eV. Deconvolution of the Ti 2p region revealed three distinct chemical states, identified as Ti‐C, Ti(II), and Ti(IV) [40, 41]. Furthermore, the high‐resolution O 1s and F 1s spectra confirmed the presence of the three key terminal groups on the Ti_3_C_2_T_x_ MXene: (i) oxide (─O at 530.91 eV), (ii) hydroxide (─OH at 532.41 eV), and (iii) fluoride (─F at 685.85 eV) [42, 43, 44]. Complementary analysis identified the structural signatures of the Nafion binder, as evidenced by the presence of CF_x_ moieties (observed in the C 1s region between 290.76 and 293.34 eV and the F 1s region at 689.11 eV) and sulfonate coordination (S 2p region). These results collectively demonstrate the effective formation of a cohesive MXene@Nafion composite [45, 46, 47].

### 2.2 Optimization of the Electrochemical Parameters

#### 2.2.1 Optimization of MXene Modification Methods

The procedure for modifying the GCE with MXene was systematically optimized. To improve the adhesion and stability of the nanomaterial layer, Nafion and chitosan were each tested as binding agents, and their effects on electrode performance were compared. As shown in Figure S2a, differential pulse voltammetry (DPV) analysis of a 10 µg mL^−1^ METH solution revealed that the MX@Nf‐modified GCE displayed an improved electrochemical response compared to both the MXene@chitosan modified GCE and the MXene/chitosan modified GCE. Furthermore, the concentration of MXene was optimized, with the results presented in Figure S2b identifying 1.5 mg mL^−1^ as the optimal concentration for achieving the best sensor performance.

#### 2.2.2 Optimization of the Electrolyte System

**FIGURE 3:**
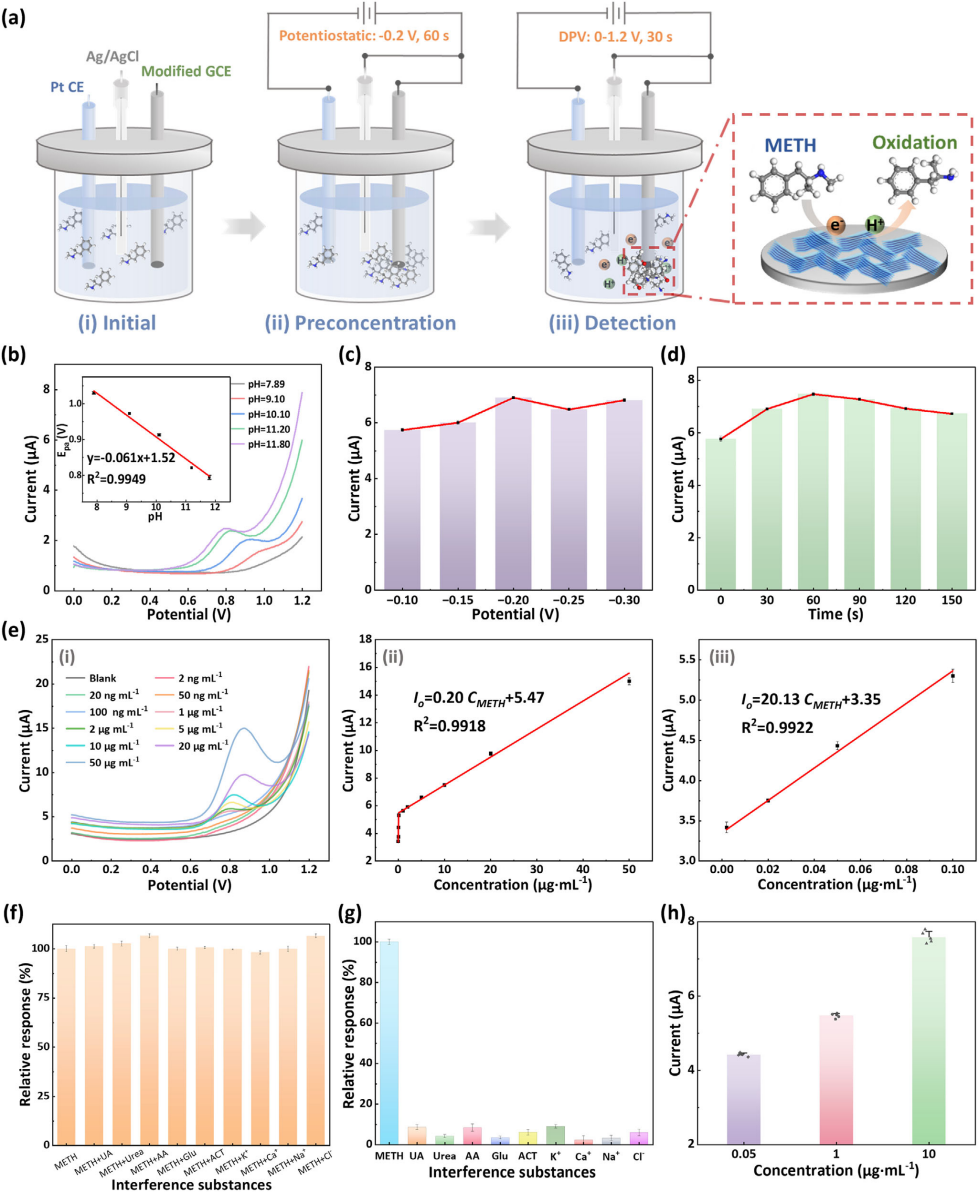
(a) Schematic illustration of the METH detection procedure. (b) DPV responses of 10 µg mL^−1^ METH at various pH values (7.89–11.80). Inset: linear relationship between oxidation peak potential and pH (*n* = 3, mean ± SD). Optimization of (c) preconcentration potential and (d) preconcentration time (*n* = 3, mean ± SD). (e) (i) DPV responses for increasing concentrations of METH. (ii, iii) Corresponding calibration curve showing the linear relationship between oxidation peak current and METH concentration (*n* = 3, mean ± SD). (f) Anti‐interference capability and (g) selectivity of the sensor against common interferents (*n* = 3, mean ± SD). (h) Repeatability of the sensor at different concentrations (*n* = 5, mean ± SD).

Four supporting electrolytes with different ionic compositions were selected to evaluate the detection performance of the MX@Nf‐modified GCE: NaOH, a NaOH‐NaCl mixture, a NaOH‐Na_2_HPO_4_ mixture, and Britton‐Robinson (BR) buffer, with all solutions adjusted to pH 11. The DPV results (Figure S2c) confirmed the detectability of METH across all electrolyte systems, with the sensor exhibiting the highest response in BR buffer. The pH dependence of METH oxidation was then investigated within the BR buffer across a pH range of 7.89–11.80 to establish the optimal detection conditions. As shown in Figure 3b, a comparative analysis of the DPV results revealed that the sensor demonstrated a well‐defined oxidation peak and an enhanced current output at pH 11.20. Based on a holistic evaluation of reaction condition mildness, current response magnitude, and the effective suppression of the oxygen evolution reaction, all subsequent electrochemical investigations were conducted in BR buffer maintained at this optimized pH of 11.20.

#### 2.2.3 Optimization of DPV Scanning Parameters

The DPV operational parameters, including pulse duration (t_pulse_) and pulse potential (E_pulse_), were optimized to obtain the optimal sensor response. As demonstrated in Figure S2d, a t_pulse_ of 20 ms was selected because shorter durations amplified capacitive current contributions, inducing substantial nonlinear baseline distortion. While increasing the E_pulse_ progressively enhanced the DPV current intensities (Figure S2e), excessive values (>50 mV) caused peak distortion due to kinetic limitations. Therefore, an E_pulse_ of 50 mV was adopted to balance signal amplification with peak resolution. Notably, introducing a preconcentration step prior to the METH oxidation scan was found to improve the current responses. This enhancement is attributed to the potential‐driven accumulation of METH molecules near the electrode interface. The optimization of the preconcentration potential and duration (Figure 3c,d) identified −0.2 V and 60 s as the optimal parameters for subsequent detection.

### 2.3 Analytical Performance

#### 2.3.1 Linearity

The sensor's response to METH was evaluated using standard solutions prepared through serial gradient dilution. Electrochemical experiments were conducted in BR buffer (pH 11.20) under the optimized operational parameters detailed in the protocol in Figure 3a. The total analysis time for the quantification of METH was 90 s, comprising a 60 s accumulation step followed by a DPV scan. As shown in Figure 3e, the MX@Nf‐modified GCE exhibited a concentration‐dependent anodic current response in the DPV measurements. The oxidation current (*I_O_*) displayed distinct piecewise linear relationships with METH concentration (*C_METH_*) across two ranges: 2–100 ng mL^−1^ (*I_O_* (µA) = 20.13 *C_METH_* (µg mL^−1^) + 3.35,  R^2^ = 0.9922) and 0.1–50 µg mL^−1^ (*I_O_* (µA) = 0.20 *C_METH_* (µg mL^−1^) + 5.47,  R^2^ = 0.9918). The dual‐linear behavior reflects a kinetic transition common in electrochemical sensing, where the rate‐limiting step shifts from interfacial reaction control at low concentrations to mass‐transfer control at higher concentrations [48]. The sensor attained a detection limit of 1.99 ng mL^−1^ (based on a signal‐to‐noise ratio S/N = 3), confirming its high analytical competence for the determination of METH.

#### 2.3.2 Anti‐Interference Performance

The specificity of the MX@Nf‐modified sensor was evaluated against common physiological interferents to assess its suitability for biofluid analysis. The targeted interferents comprised ascorbic acid (AA), uric acid (UA), urea, glucose (Glu), acetaminophen (ACT), and major electrolyte ions (K^+^, Na^+^, Ca^2+^, Cl^−^). Experiments were conducted with a fixed METH concentration of 5 µg mL^−1^, while the interferents were introduced at a 10‐fold higher concentration (50 µg mL^−1^). As evidenced by the anti‐interference results in Figure 3f and the selectivity analysis in Figure 3g, all interferents elicited signal perturbations that remained below the 10% threshold. This demonstrates the sensor's strong resistance to interference in complex matrices.

#### 2.3.3 Repeatability and Consistency

The sensor's repeatability was assessed through five consecutive measurements of varying METH concentrations (50 ng mL^−1^, 1, and 10 µg mL^−1^) using a single MX@Nf‐modified GCE. As shown in Figure 3h, the current responses exhibited favorable reproducibility, with relative standard deviations (RSD) of 0.96%, 1.12%, and 2.06% for each concentration level, respectively. To assess batch consistency, three independently fabricated sensors underwent identical electrochemical measurement at METH concentrations of 0.5, 1, and 5 µg mL^−1^. The inter‐device RSD values across all tested concentrations remained below the 5% threshold (Table S2), statistically confirming the reliability of the manufacturing protocol. These collective findings demonstrate that the sensor possesses satisfactory operational repeatability and good batch‐to‐batch consistency.

### 2.4 Sensing and Detection Mechanism of METH

To better understand the electrochemical reaction of METH at the electrode surface and MXene's role in signal amplification and sensitivity enhancement, we systematically analyzed the detection mechanism by combining experimental characterization with theoretical simulations.

#### 2.4.1 Electrochemical Behavior of METH at MX@Nf‐Modified GCE

We first investigated the electrochemical behavior and detection mechanism of METH using cyclic voltammetry (CV) by immersing the sensor in a 10 µg mL^−1^ METH solution. As depicted in Figure S3a, the introduction of METH resulted in a distinct oxidation peak at approximately 0.85 V during the anodic sweep. Compared to the bare GCE, the MX@Nf nanomaterial clearly accelerates electron transfer kinetics and enhances the electrochemical response. The absence of a corresponding reduction peak in the reverse scan indicates the irreversible nature of this electrochemical oxidation process.

**FIGURE 4:**
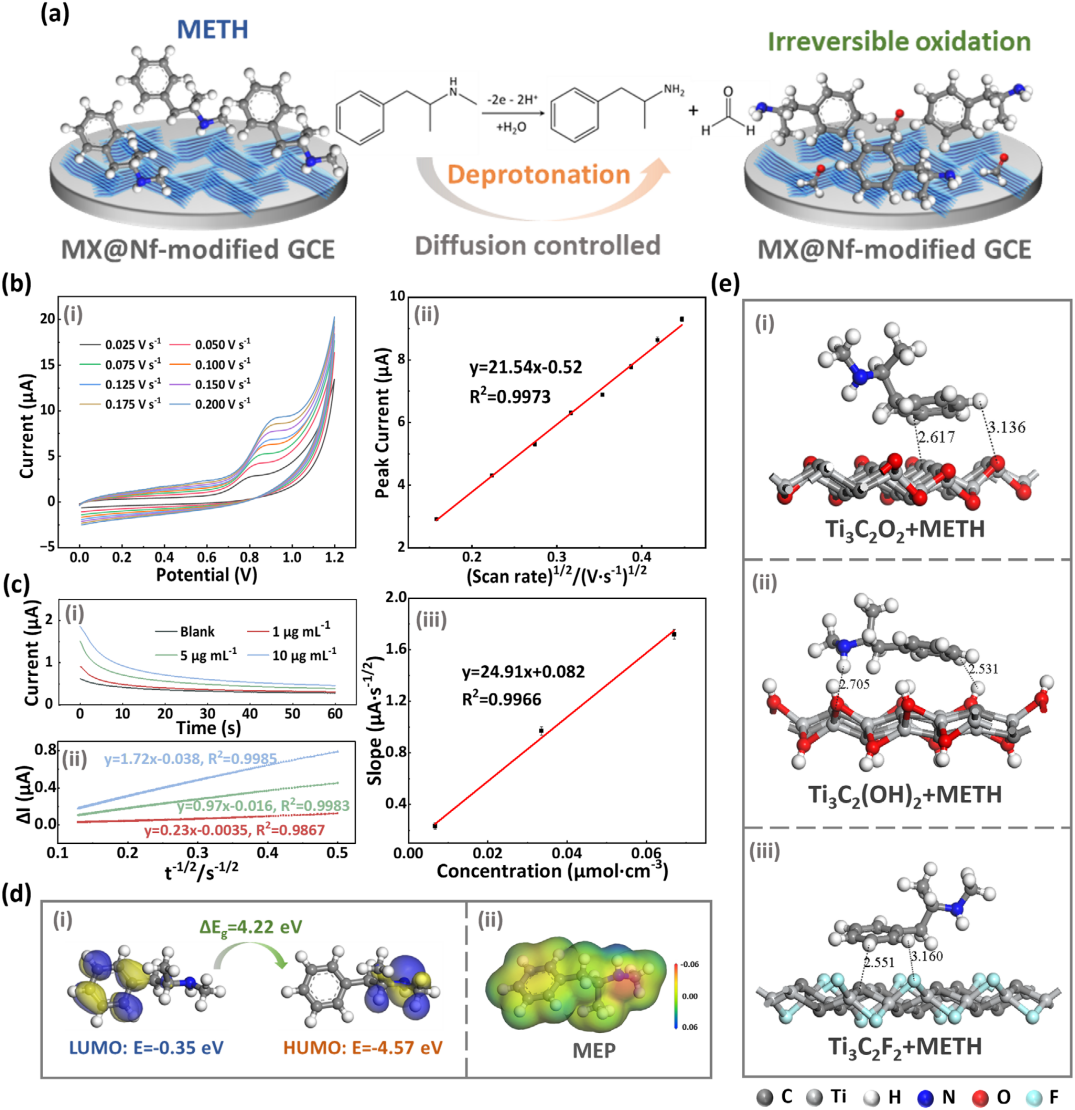
(a) Proposed electrochemical oxidation mechanism of METH on the MX@Nf‐modified GCE. (b) (i) CV responses of 10 µg mL^−1^ METH at different scan rates (0.025–0.20 V s^−1^). (ii) Linear relationship between the anodic peak current and the square root of the scan rate (*n* = 3, mean ± SD). (c) (i) Chronoamperometric responses at +0.85 V with different METH concentrations (0–10 µg mL^−1^). (ii) Linear relationship of ∆*I* versus *t* ^−1/2^, (*n* = 3, mean ± SD). (iii) Linear relationship between the slope values and METH concentration (*n* = 3, mean ± SD). (d) Computational analysis of the METH molecule: (i) spatial distributions of the LUMO and HOMO, and (ii) molecular electrostatic potential map. (e) Final configurations from molecular simulations depicting METH interactions with different MXene surfaces.

To elucidate the oxidation kinetics, CV measurements were performed at scan rates ranging from 25 to 200 mV s^−1^. As illustrated in Figure 4b, the oxidation peak current (*I_pa_*) increased with the scan rate (*v*), accompanied by a concomitant positive shift in potential. A linear correlation was observed between *I_pa_* and the square root of the scan rate (*v* ^1/2^), described by the regression equation: *I_pa_* (µA) = 21.54 *v* ^1/2^ (V·s^−1^) ‐ 0.52 (R^2^ = 0.9973). This linear relationship, supported by a corresponding linear plot of logarithmic peak current (log *I_pa_*) versus logarithmic scan rate (log *v*), yielding a slope of 0.55 (Figure S3b), provides strong evidence for a diffusion‐controlled electrochemical process.

We then analyzed the relationship between oxidation peak potential (*E_pa_*) and scan rate to elucidate the charge transfer process. A linear dependence of *E_pa_* on the logarithm of the scan rate was observed, expressed as *E_pa_* (V) = 0.046 ln *v* (V·s^−1^) + 1.02 (R^2^ = 0.9913) (Figure S3c). Given the irreversible nature of the reaction, the Laviron equation (Equation 1) was employed to determine the number of electrons transferred (*n*).

(1) $$E_{pa}=E^{0}+\frac{RT}{\alpha nF}\;\ln\left(\frac{RTk_{s}}{\alpha nF}\right)+\frac{RT}{\alpha nF}\;\ln\;v$$

here, *E^0^* represents the standard electrode potential, *k_s_* is the standard rate constant, and *R*, *T*, and *F* represent the gas constant, temperature, and Faraday's constant, respectively. Based on the methodology described in Reference [49] and assuming a typical charge transfer coefficient *α* of 0.3, the electrochemical oxidation of METH was determined to involve a two‐electron transfer process.

What's more, on the basis of this diffusion‐controlled characteristic, the diffusion coefficient (*D*) of METH was calculated using chronoamperometry. Measurements were conducted in BR buffer (pH = 11.20) containing METH at 0, 1, 5, and 10 µg mL^−1^, with the working potential held at 0.85 V (vs Ag/AgCl). As shown in Figure 4c (i), the current response (*I*) increased with METH concentration (*C_METH_*). The Cottrell equation (Equation 2) describes the current responses for a diffusion‐controlled process:

(2) $$I=\frac{\mathrm{nFAC}D^{1/2}}{\pi^{1/2}t^{1/2}}=K\cdot t^{-1/2}$$

where *n* represents the number of electrons transferred, *A* is the electrode area, *t* is the testing time, and *K* is the slope [48, 50]. In practice, interference currents introduce a constant intercept *B*, making the observed relationship *I* = *K*·*t* ^−1/2^ + *B*. To accurately isolate the diffusion‐controlled *K* and determine *D*, chronoamperometry was performed at multiple concentrations. The background current (0 µg mL^−1^) was subtracted to obtain the net current (∆*I*). Linear regression of ∆*I* versus *t* ^−1/2^ (Figure 4c (ii)) yielded the slope *K* for each METH concentration. A plot of these slopes against *C_METH_* confirmed a direct proportional relationship, *K* ∝ *C* (Figure 4c (iii)), validating the application of Equation (2). The diffusion coefficient *D*, calculated from this proportionality, was 1.05 × 10^−5^ cm^2^ s^−1^.

To further investigate proton involvement in the oxidation, we analyzed DPV responses of METH solutions across a pH range of 7.89 to 11.80. As shown in Figure 3b, alkaline conditions significantly enhanced METH oxidation efficiency, characterized by a progressive shift of the oxidation peak toward lower potentials and a concurrent increase in peak current. This observed electrochemical behavior aligns well with the documented pKa value of METH [26, 50, 51]. A linear correlation was established between the oxidation peak potential (*E_pa_*) and pH (inset of Figure 3b), expressed as: *E_pa_* (V) = ‐0.061 *pH* + 1.52 (R^2^ = 0.9949). The slope of −0.061 V pH^−1^ closely matches the theoretical Nernstian value of −0.059 V pH^−1^, suggesting that the oxidation mechanism involves an equal number of protons and electrons [52, 53].

To identify the molecular sites responsible for electrochemical oxidation, we performed first‐principles density functional theory (DFT) simulations to analyze METH's electron density distribution and frontier molecular orbitals. As revealed by the highest occupied molecular orbital (HOMO) distribution (Figure 4d (i)), electron density is predominantly localized on the aliphatic secondary amine group (‐NH) and the adjacent atoms. Moreover, atomic reactivity analysis via the Fukui function (Figure S4) indicated that the nitrogen atom in the ‐NH group has the highest electrophilic attack index, confirming its superior reactivity and oxidation susceptibility. Additionally, the molecular electrostatic potential (MEP) map (Figure 4d (ii)) demonstrates that the nitrogen atom on the ‐NH group exhibits a region of lower electrostatic potential (red, indicating electronegative character), identifying it as an electron‐rich center prone to oxidation. Collectively, these computational results confirm that the electrochemical oxidation of METH occurs predominantly at the ‐NH group [28]. The DFT calculations yielded energies of −4.57 eV for the HOMO and −0.35 eV for the lowest unoccupied molecular orbital (LUMO). The energy difference, or HOMO‐LUMO gap (Δ*E_g_* = *E_LUMO_*− *E_HOMO_* = 4.22 eV), governs the ease of molecular excitation; a smaller gap facilitates greater excitability, indicating high chemical reactivity and low kinetic stability for the METH molecule [54, 55]. Furthermore, the experimental UV–vis absorption spectrum of METH (Figure S5) exhibited a maximum absorption wavelength (*λ*) at 257 nm. According to the photon energy formula (Equation 3), where *h* represents the Planck constant and *c* is the speed of light, the optical bandgap derived from this *λ* is 4.56 eV. This value closely approaches the theoretical minimum gap, demonstrating good agreement between experimental results and computational predictions.

(3) $$E=\frac{hc}{\lambda }$$

Finally, to identify the reaction products, the solutions before and after the electrochemical reaction were analyzed using mass spectrometry (MS). The signals corresponding to the parent ions and their fragmentation patterns are shown in Figure S6. A comparison of the MS signals of the oxidation product with those of amphetamine, coupled with the previously reported oxidation behavior of aliphatic secondary amines [26, 27, 56, 57], led us to conclude that the primary product of the electrochemical oxidation is amphetamine, with the concomitant generation of formaldehyde (Figure S6c).

In summary, by integrating experimental findings with theoretical simulations, we propose that the electrochemical oxidation of METH occurs primarily at the secondary amine group, which exhibits the highest susceptibility to oxidation and demonstrates enhanced reactivity under alkaline conditions. As illustrated in Figure 4a, the proposed mechanism involves the initial deprotonation of METH followed by a two‐electron transfer process, ultimately converting the secondary amine to a primary amine and generating formaldehyde as a byproduct.

#### 2.4.2 Signal Enhancement Mechanism of MXene

To further elucidate the molecular interactions at the sensing interfaces, theoretical calculations were performed to analyze the behavior of METH on different electrode surfaces. Structural models of the METH molecule, a bare glassy carbon (GC) substrate, and three MXene variants (Ti_3_C_2_O_2_, Ti_3_C_2_(OH)_2_, and Ti_3_C_2_F_2_) were constructed, as presented in Figures S7 and S8. The final simulated configurations of METH molecules interacting with both the MXene and GC surfaces are shown in Figure 4e and Figure S9.

The analysis reveals that the METH molecule can form hydrogen‐bonding interactions with all three functionalized MXene surfaces. The minimum distances between METH and the different surfaces, determined by measuring the shortest atom‐to‐atom separations, are summarized in Table S3. As depicted in Figure 4e, the primary interaction on the Ti_3_C_2_O_2_ surface occurs via C─H⋯O, with a distance of 3.136 Å. On the Ti_3_C_2_(OH)_2_ surface, hydrogen bonds are formed between hydroxyl (─OH) groups and H atoms of METH, with bond lengths of 2.924 and 2.531 Å. For Ti_3_C_2_F_2_, the dominant hydrogen‐bonding interaction is C─H⋯F, with a corresponding distance of 3.160 Å. A comparison of the shortest distances shows that METH is closer to all MXene surfaces than to bare GC. This is attributed to MXene's functional groups, which expand the effective interaction region, restrict METH migration, and provide more interaction sites, thereby enhancing productive encounters.

The binding energies (*E_be_*) between METH and the different surfaces were calculated using Equation (4) [58]. As demonstrated in Table 1, all three forms of MXene exhibit stronger binding affinity toward METH than the bare GC surface, indicating stronger interfacial interactions. Notably, Ti_3_C_2_O_2_ shows the highest binding energy (−0.48 eV) with METH, demonstrating the superior affinity of its oxygen functional groups and theoretically identifying it as the most effective modifier for METH oxidation and detection. The calculated binding energy primarily originates from non‐covalent interactions dominated by weak forces, particularly hydrogen bonding. The formation and increased number of hydrogen bonds between METH and MXene enhance the strength of these non‐covalent interactions, consequently improving the stability of the entire system.

(4) $$E_{\mathrm{be}}=E_{\mathrm{total}}-\left(E_{\mathrm{METH}}+E_{Ti_{3}C_{2}T_{2}}\right)$$

**TABLE 1:** Binding energies of METH on different sensing surfaces.

	Ti_3_C_2_O_2_‐METH	Ti_3_C_2_(OH)_2_‐METH	Ti_3_C_2_F_2_‐METH	GC‐METH
*E_be_*	−0.48 eV	−0.25 eV	−0.32 eV	−0.028 eV

In summary, these simulation studies reveal that the abundant functional groups on MXene surfaces provide enhanced affinity sites for electrochemical reactions. The constructed MXene sensitive layers effectively reduce the distance between METH molecules and the sensing interface while promoting the formation of weak hydrogen bonds. This dual effect significantly increases both the frequency and strength of molecular interactions at the analyte‐interface, thereby establishing a solid theoretical basis for the amplified electrochemical response signals observed experimentally.

### 2.5 Applications in Real Samples

**FIGURE 5:**
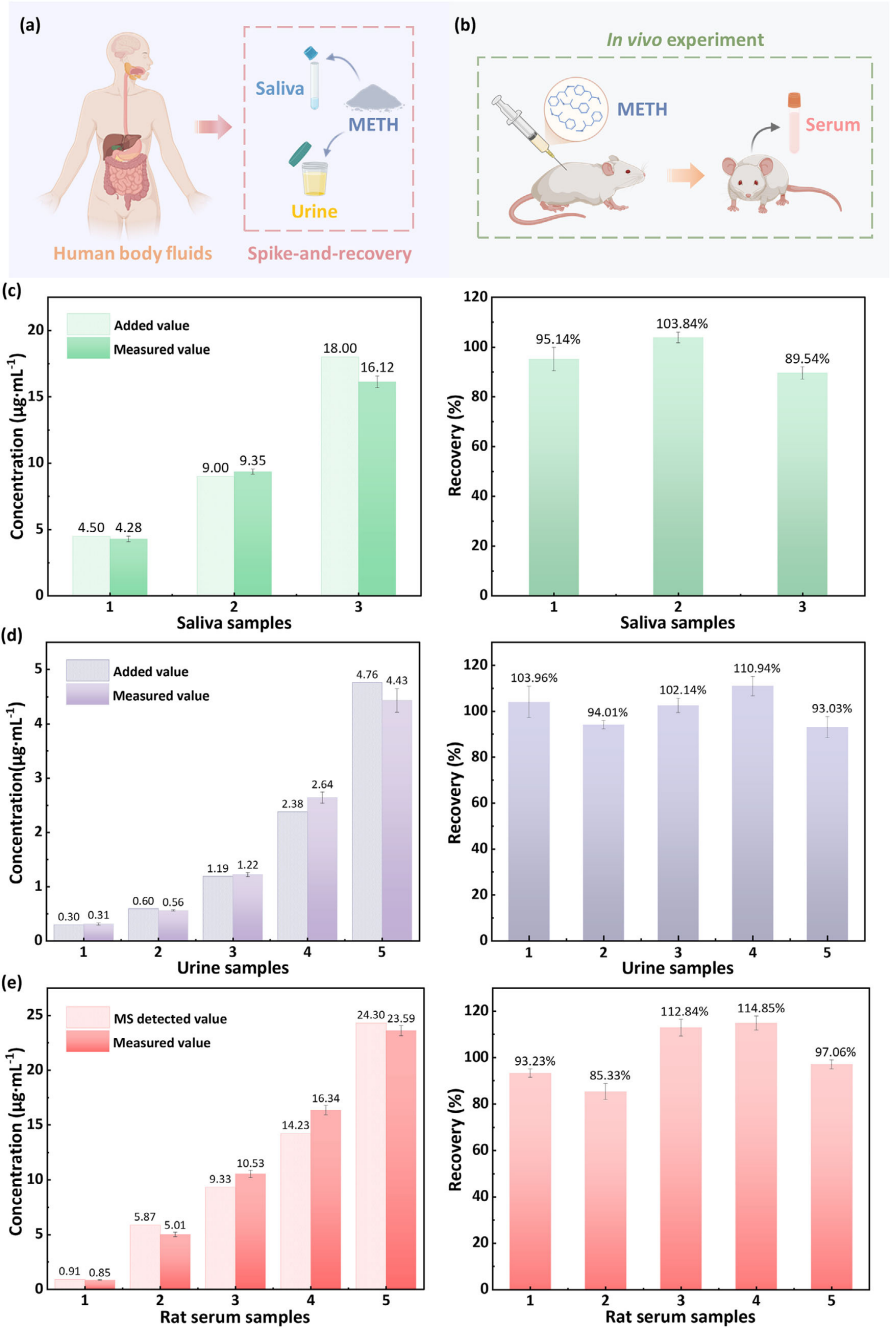
Applications in real sample detection. (a) Schematic of the spike‐and‐recovery tests performed in artificial saliva and urine. (b) Schematic diagram of the in vivo experimental procedure: intravenous METH administration to rats followed by serial blood collection for serum analysis. Recovery analysis of METH in (c) artificial saliva, (d) artificial urine, and (e) rat serum samples with comparative data from the reference MS method (*n* = 3, mean ± SD).

The application potential of the developed sensor for METH monitoring was evaluated through quantitative analyses in both simulated and in vivo biological matrices. First, artificial saliva and urine matrices were employed to simulate physiological environments, as shown in Figure 5a. Spike‐and‐recovery assays demonstrated consistent analyte recovery rates ranging from 89.54% to 110.94% (Figure 5c,d). These results confirm that matrix interference is negligible and validate the sensor's feasibility and reliability for the quantification of METH in human‐mimetic environments.

For in vivo validation, we analyzed serum samples from Sprague Dawley (SD) after intravenous METH administration (Figure 5b). Parallel measurements were performed with the MX@Nf‐modified sensor and the standard MS method for comparison. As illustrated in Figure S10, three distinct voltametric peaks were observed in the rat serum samples during direct detection with the MX@Nf‐modified GCE. In addition to the characteristic oxidation peak of METH (>0.8 V), additional signals were detected at approximately 0.18 and 0.51 V, which are primarily attributed to the oxidation of endogenous species such as dopamine and glucose [59, 60]. The higher oxidation potential of METH enables its selective detection, ensuring minimal interference from these endogenous electroactive species during quantification. As illustrated in Figure 5e, the results from the sensor correlated well with the MS reference data, showing accuracies between 85.33% and 114.85%. This close agreement establishes the sensor's reliability for real‐time monitoring applications in complex biological samples.

### 2.6 Comparison with Other Works

This study presents an electrochemical sensor for METH detection based on electrochemical oxidation and enhanced by a MXene‐modified nano‐sensing film. As illustrated in Table 2, a comparison with other electrochemical sensors that employ nanomaterial modifications or surface treatments for METH oxidation demonstrates that the developed sensor offers competitive analytical performance. It exhibits a low detection limit alongside a wide linear detection range. Furthermore, the detection performance of our sensor complies with the on‐site preliminary screening standards for METH established in China and the United States (minimum: 50 ng mL^−1^), and also reaches the theoretical concentration thresholds necessary for confirming METH intake (minimum: 5 ng mL^−1^) [61, 62, 63]. Notably, whereas conventional confirmatory methods (e.g., chromatography‐mass spectrometry) involve complex, multi‐step protocols with total analysis times often exceeding 30 min, our sensor delivers a quantitative result within 90 s. This timeframe is also advantageous compared to immunoassay‐based sensors, which typically require a 10–20 min incubation step [64, 65]; our direct oxidation approach is incubation‐free. By combining quantitative capability with a sub‐two‐minute analysis, our method achieves a critical balance for on‐site screening, making it a promising tool for rapid field‐deployable applications.

**TABLE 2:** Comparison of the analytical performance of this work with other electrochemical sensors for METH detection modified with different nanomaterials.

Sensitive interfaces	Detection methods	LOD (ng mL^−1^)	Linear range (µg mL^−1^)	Real samples	Refs.
AgNDs/CNOs/GCE^a)^	DPV	4.48	0.015–8.94	Serum, urine	[50]
SPE/MWCNTs‐Nf/GNPs^b)^	SWASV^c)^	1.10	3.7 × 10^−3^–1.8 × 10^−2^, 0.55–9.20	/	[51]
	EIS^d)^	0.056	0.21 × 10^−3^–0.50 × 10^−3^		
GCE/MWCNT/Au‐NPs‐HS‐SiO_2_/Fe_3_O_4_ ^e)^	SWV^f)^	2.39	7.46 × 10^−3^–7.46	Urine	[52]
Electrochemically pretreated pencil graphite electrode	DPV	7.46	0.011–8.06	Urine, serum, seized drugs	[66]
CeO_2_ NP/rGO‐GCE^g)^	SWV	1305.82	3.73–24.86	Plasma	[67]
Graphene modified SPCE	SWV	44.77	0.15–74.62	Seized drugs, water samples	[68]
Carbon materials modified GCE	DPV	44.77	0.15–17.91	Household surfaces	[69]
MXene@Nafion modified GCE	DPV	1.99	2 × 10^−3^–50	Urine, saliva, serum	This work

^a^ Silver nanodendrites on nanodiamond‐derived carbon nano‐onions modified glassy carbon electrode;

^b^ gold nanoparticle and multiwalled carbon nanotube‐Nafion modified screen‐printed electrode;

^c^ square wave anodic stripping voltammetry;

^d^ electrochemical impedance spectroscopy;

^e^ glassy carbon electrode modified with amine functionalized multi‐walled carbon nanotube and gold nanoparticles linked to nanomagnetic core shells;

^f^ square wave voltammetry;

^g^ nanoceria nanoparticle‐decorated reduced graphene oxide modified glassy carbon electrode.

## 3 Conclusions

In summary, we developed a rapid, direct electrochemical method for METH detection using MXene as a sensitive interface material. Through integrated theoretical and experimental analyses, we elucidated MXene's pivotal role in signal amplification. The layered architecture of MXene accelerates charge‐transfer kinetics, while its rich surface functional groups enhance both the probability and strength of interactions between METH molecules and the electrode. This synergistic effect improves molecular contact through structural compatibility and directly drives the amplified electrochemical responses. These findings establish a robust theoretical framework for sensor design and underscore the significant potential of MXene‐based interfaces in advancing portable analytical technologies.

Under optimized conditions, the sensor achieved sensitive METH detection with a wide linear detection range (2 ng mL^−1^–50 µg mL^−1^), a rapid response (<90 s), and a low detection limit (1.99 ng mL^−1^). It also demonstrates strong anti‐interference capability in complex matrices, along with satisfactory operational repeatability and batch‐to‐batch consistency. Moreover, successful spike‐recovery tests in urine and saliva, coupled with the direct detection of METH in rat serum, validated the sensor's practical applicability.

Illicit drug screening is of paramount importance worldwide. This study establishes a promising and viable technological platform for the on‐site and rapid detection of METH and other illicit substances, demonstrating its potential effectiveness in law‐enforcement and public‐health surveillance. However, the current system's reliance on electrochemical workstations limits its application in true point‐of‐care settings. Future work will therefore focus on developing a portable, integrated field‐deployment system. This will involve device miniaturization, smartphone integration, expanded validation using diverse real‐world samples, and the implementation of multi‐analyte detection capabilities. Such advancements are essential to transform this sensing platform into a robust, practical tool for on‐site drug monitoring.

## 4 Experimental Section

### 4.1 Materials

MXene (Ti_3_C_2_T_2_, T═O, OH, and F) was procured from Xinxi Technology Co., Ltd. (Foshan, China). NaOH, Na_2_HPO_4_, H_3_PO_4_, CH_3_COOH, H_3_BO_3_, CaCl_2_, NaCl, and KCl were obtained from Sinopharm Chemical Reagent Co., Ltd. (Shanghai, China). Ascorbic acid, uric acid, urea, glucose, and acetaminophen were supplied by Aladdin Biochemical Technology Co., Ltd. (Shanghai, China). A Nafion perfluorinated resin solution was purchased from Shengernuo Technology (Suzhou, China). Standardized artificial saliva (formulated according to ISO/TR 10271) and artificial urine were acquired from Yuanye Bio‐Technology Co., Ltd. (Shanghai, China). METH standards were sourced from an authorized law enforcement agency with formal approval for use in scientific research. All aqueous solutions were prepared using deionized water. Unless otherwise specified, all experiments were conducted at an ambient temperature of 25°C.

### 4.2 Fabrication of the Sensor

The sensor was fabricated through a drop‐casting procedure as illustrated in Figure 2a. First, MXene suspensions of varying concentrations were prepared by dispersing clay‐form MXene powder in deionized water under continuous sonication. To improve adhesion, a 0.5% Nafion solution (25 µL) was incorporated into the MXene suspension (500 µL). This binder strengthens the interfacial bonding and mechanical integrity between the MXene nanostructures and the electrode substrate, which was critical for maintaining a stable and functional sensing surface. Following thorough vortex agitation and ultrasonic dispersion to achieve a homogeneous mixture, a 10 µL aliquot of the resulting suspension was carefully drop‐cast onto the surface of a pre‐cleaned GCE. The MXene@Nafion modified GCE was obtained after drying at room temperature for 12 h to ensure the formation of a stable and uniform film.

For comparative analysis, MXene@chitosan modified GCE and MXene/chitosan modified GCE were also prepared following a similar protocol; the detailed preparation steps for these control electrodes are provided in the Supporting Information.

### 4.3 Instruments and Equipment

The nanoscale morphology of the MXene@Nafion nanocomposites was examined with a Zeiss Sigma 300 scanning electron microscope (Carl Zeiss, Germany). Transmission electron microscopy was performed on a JEM‐F200 instrument (JEOL, Japan). Elemental distribution was mapped using an Oxford Xplore energy‐dispersive spectrometer (Oxford Instruments, UK). Surface chemical composition and bonding states were analyzed with a Thermo Scientific K‐Alpha X‐ray photoelectron spectrometer (Thermo Fisher Scientific, USA). UV–vis absorption spectra were recorded on an OSE‐260‐06 full‐wavelength spectrophotometer (Tiangen Biotech, China). Mass‐spectrometry data were acquired using a LCMS‐8060 triple‐quadrupole‐liquid‐chromatograph mass spectrometer (Shimadzu Corporation, Japan) and a C3001‐Sci miniature mass spectrometer (Purspec Technologies, China) for reference analysis.

### 4.4 Theoretical Computational Methods

The bare GCE surface structure was modeled using classical molecular mechanics as implemented in the Forcite module. An amorphous carbon unit cell was constructed and subsequently subjected to a high‐temperature carbonization simulation process. Molecular dynamics relaxation was performed in the NVT ensemble at 3000 K for 50 ps to achieve thermal equilibration. The system then underwent a controlled annealing process from 3000 to 300 K, followed by geometry optimization. The most thermodynamically stable configuration obtained was selected for all subsequent analysis.

First‐principles density functional theory computations were carried out using the Cambridge Sequential Total Energy Package (CASTEP) module to investigate the interfacial interactions and the underlying detection mechanism of METH on both the glassy carbon and MXene surfaces. Various MXene crystals (Ti_3_C_2_O_2_, Ti_3_C_2_(OH)_2_, and Ti_3_C_2_F_2_) were modeled in the *P* $\bar{3}$ *m*1 space group (No. 164). Their surfaces were generated through cleavage along the (110) crystallographic plane followed by supercell expansion [70, 71]. Following geometric optimizations of all individual components, molecular simulations and interaction energy calculations were performed to quantitatively assess the strength and nature of the interfacial interactions.

### 4.5 Electrochemical Evaluation

All electrochemical measurements were performed using a PalmSens4 portable potentiostat (PalmSens BV, Netherlands) configured in a standard three‐electrode setup, using a platinum counter electrode and an Ag/AgCl (3 m KCl) reference electrode. The electrochemical behavior of METH was primarily investigated by cyclic voltammetry with a potential window spanning from 0 to +1.2 V (vs Ag/AgCl). Chronoamperometric analysis was conducted by applying a constant potential of +0.85 V for 60 s to evaluate the diffusion characteristic of METH. For the quantitative detection of METH, differential pulse voltammetry was employed under alkaline conditions over a potential range of 0–+1.2 V.

### 4.6 Real Samples Detection

The sensor's detection capability was first evaluated in artificial urine and saliva to validate its performance under physiologically relevant conditions. A series of known METH concentrations was spiked into these simulated biological fluids and measured directly using the MX@Nf‐modified sensor. The accuracy of the method was rigorously assessed through recovery studies using the standard addition approach.

Furthermore, in vivo experiments were performed using adult Sprague Dawley rats (weighing 250 ± 50 g). The animals were housed under a standard 12 h light/12 h dark cycle with ad libitum access to food and water. All animal experimental procedures were reviewed and approved by the Institutional Animal Care and Use Committee at the Shenzhen Institutes of Advanced Technology, Chinese Academy of Sciences. The experimental procedure was conducted as follows: rats were anesthetized and subjected to a catheterization procedure during which an indwelling catheter was surgically implanted into the subcutaneous jugular vein. This setup facilitated intravenous METH administration and serial blood sampling. METH was administered intravenously at a dose of 10 mg kg^−1^. Blood samples (∼ 500 µL each) were collected pre‐injection and at specific times post‐injection. Collected samples were immediately placed on ice for 30 min to clot, then centrifuged at 12,000 g for 10 min at 4°C to separate serum. The supernatant was carefully collected, and each sample was split into two aliquots: one for direct analysis with the MX@Nf‐modified sensor, and the other for confirmatory analysis by standard mass spectrometry as a reference.

### 4.7 Statistical Analysis

All quantitative experiments were conducted with a minimum of three independent replicates (n ≥ 3), as specified in the respective Figure legends. All quantitative data were presented as the arithmetic mean ± standard deviation (SD), as detailed in the Figure legends. All plotting and basic descriptive statistical calculations (mean, SD) were performed using Origin software (version 2025b, OriginLab Corporation).

## Funding

This work was supported by research funding from the National Natural Science Foundation of China (62475279), the Ministry of Science and Technology of China (2023YFF0721500), the Shenzhen Science and Technology Innovation Commission (KCXFZ20230731100901004; ZDSYS20190902093601675) and the Yunnan Technological Innovation Center of Drug Addiction Medicine (202305AK340001).

## Conflicts of Interest

The authors declare no conflicts of interest.

## Supporting information




**Supporting File**: advs73700‐sup‐0001‐SuppMat.docx.

## Data Availability

The data that support the findings of this study are available from the corresponding author upon reasonable request.
