# Supplementary material for: Rapid and Direct Detection of Methamphetamine in Biofluids using a MXene‐Enabled Electrochemical Sensor
Source: Adv Sci (Weinh). 2026 Jan 8;13(21):e21857. doi: 10.1002/advs.202521857 (PMC13073237; doi:10.1002/advs.202521857)
Supplement: Supplementary file 1 — Supporting File: advs73700‐sup‐0001‐SuppMat.docx. [file ADVS-13-e21857-s001.docx]

Supporting Information

**Rapid and Direct Detection of Methamphetamine in Biofluids using a MXene-Enabled Electrochemical Sensor**

*Ri Wang, Xiaofei Deng, Bo Chen, Jienan Shen, Wei Xu, Yingjie Zhu*, Yi Zhang*, and Hui Yang**

**Fabrication of Control Electrodes**

MXene@chitosan modified GCE (MX@CHI-modified GCE): The MXene@chitosan nanofilm was fabricated through an ultrasonic-assisted homogenization of an aqueous MXene dispersion with a 1% chitosan solution, followed by drop-casting onto the GCE surface.

MXene/chitosan modified GCE (MX/CHI-modified GCE): The polished GCE was sequentially modified by first drop-casting 3 μL of a 1% chitosan solution, followed by a drop-cast MXene dispersion.


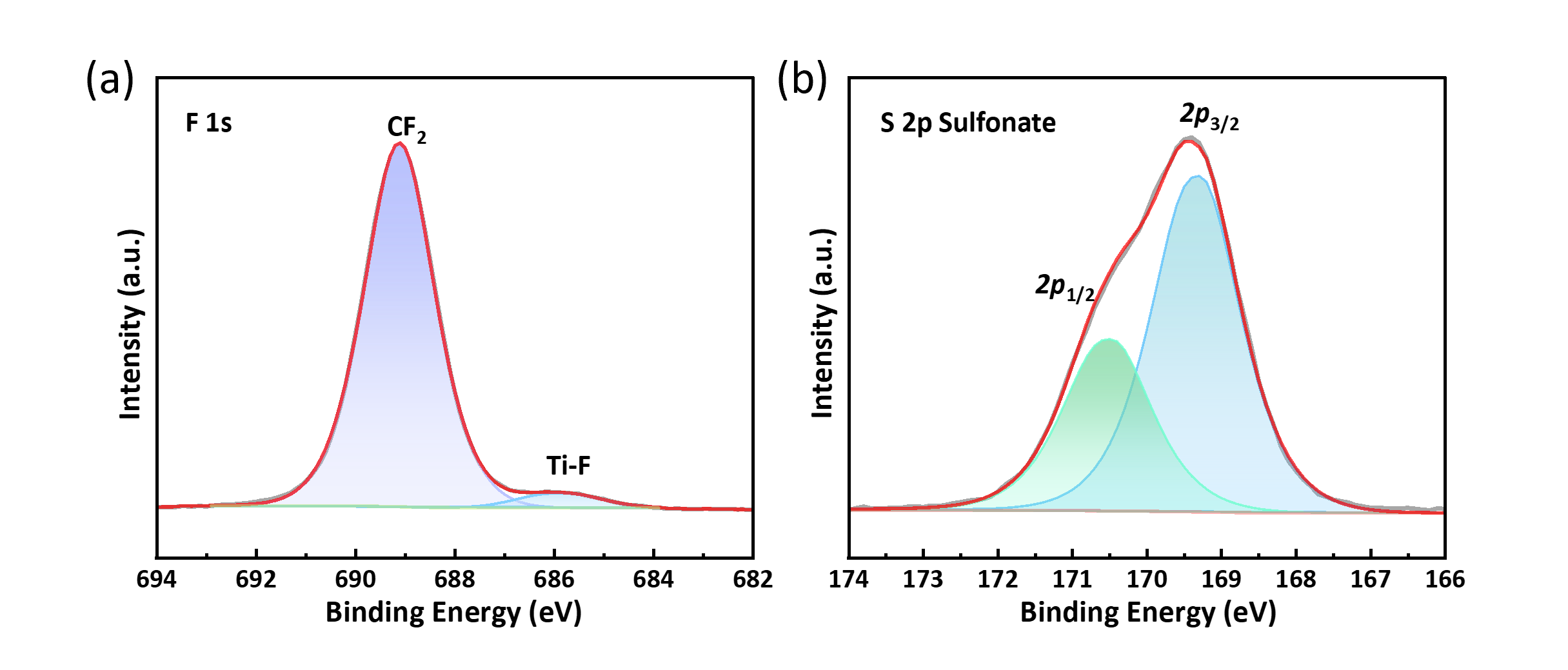


**Figure S1.** High-resolution XPS spectra of the MXene@Nafion modified GCE. (a) F 1s and (b) S 2p core-level regions.


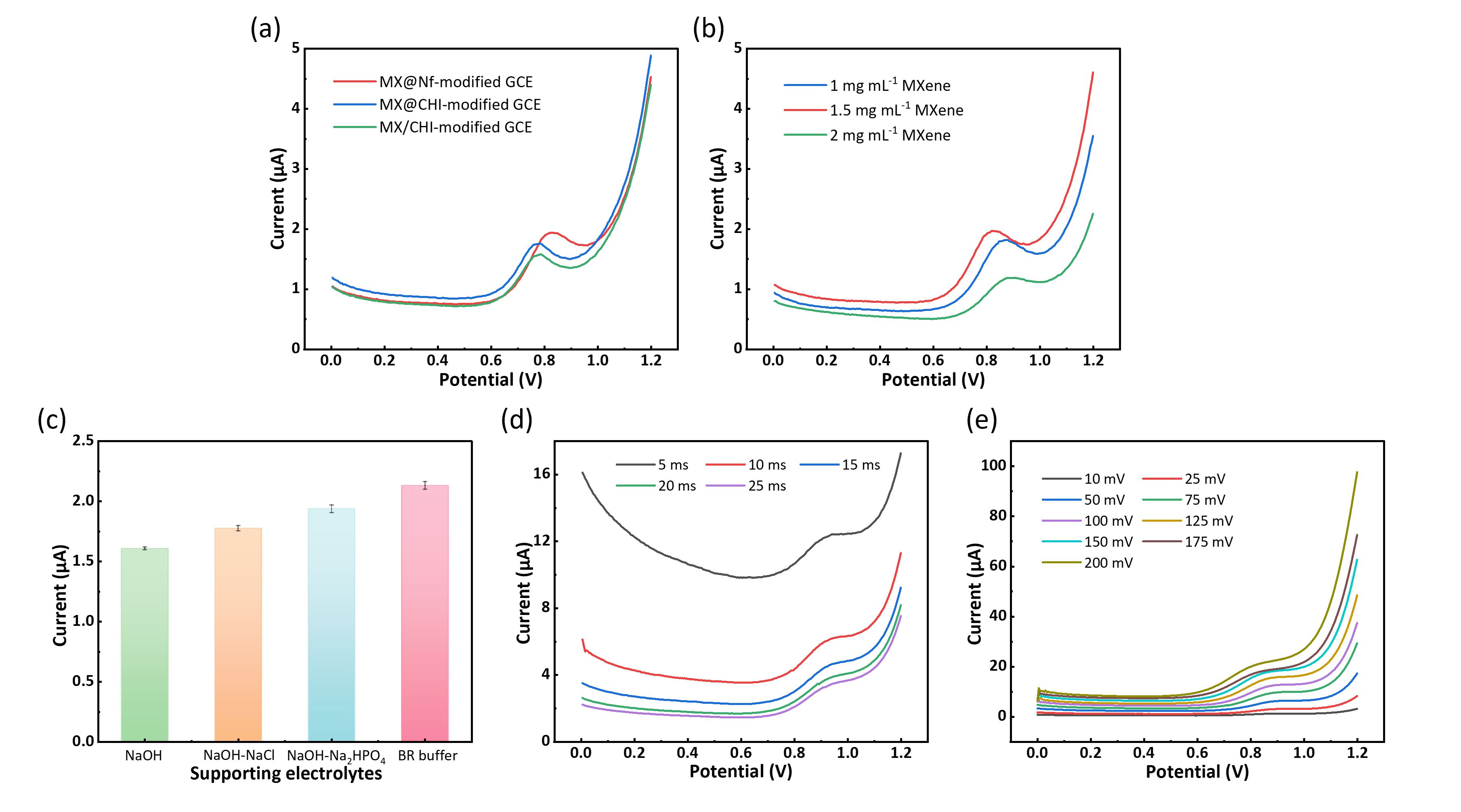


**Figure S2.** Optimization of key experimental parameters. (a) Electrode modification methods, (b) MXene concentration, (c) supporting electrolytes, (d) DPV pulse duration, and (e) DPV pulse potential.


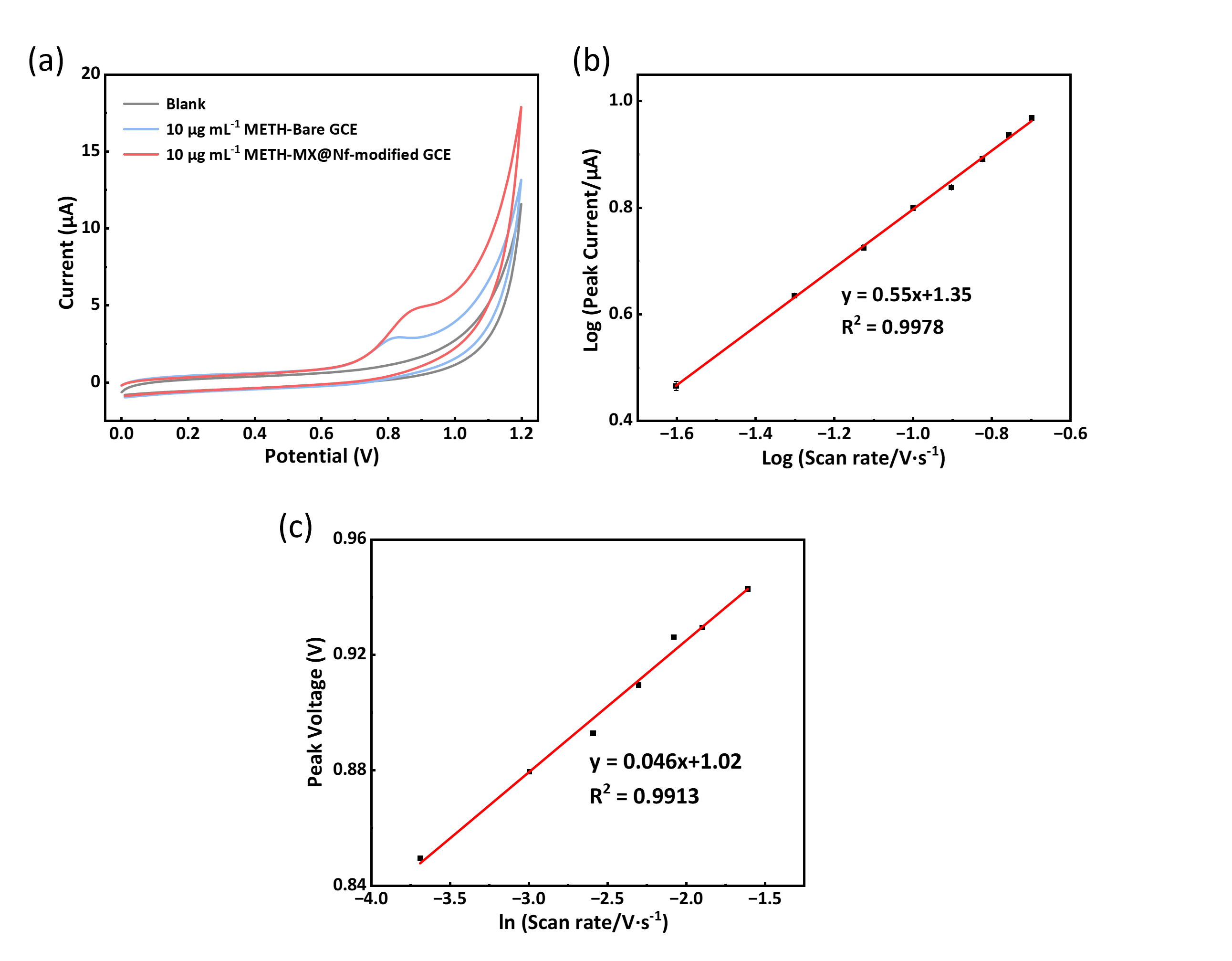


**Figure S3.** (a) CV plots of a blank solution and a 10 μg mL^-1^ METH solution on different electrodes. Linear relationship between (b) the logarithmic peak current and the logarithmic scan rate, and (c) the oxidation peak potential and the scan rate.


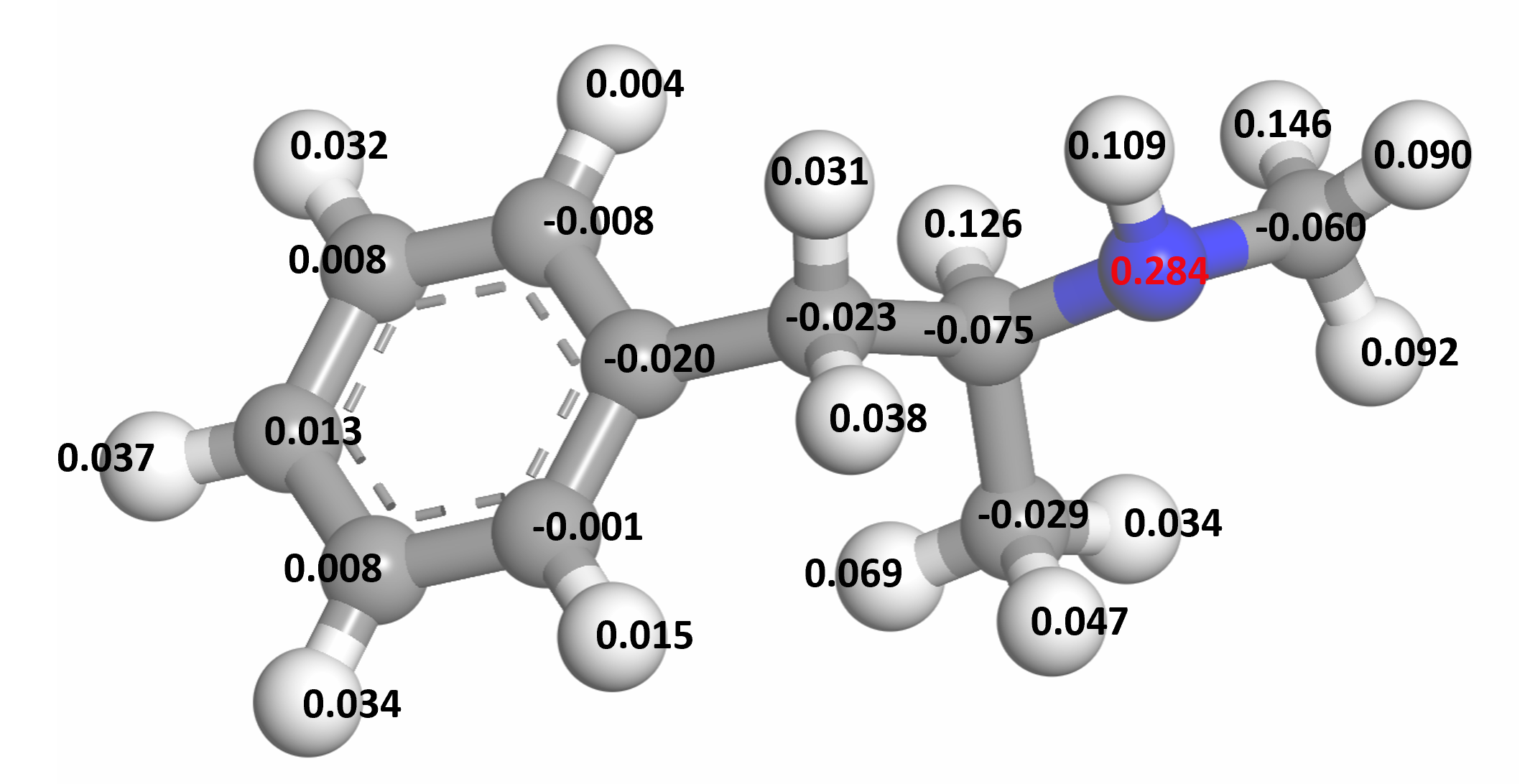


**Figure S4.** Computed Fukui function for electrophilic attack, visualizing the atomic reactivity and electrophilic reaction sites on the METH molecule. Carbon (C), hydrogen (H), and nitrogen (N) atoms are represented in dark grey, white, and dark bule, respectively.


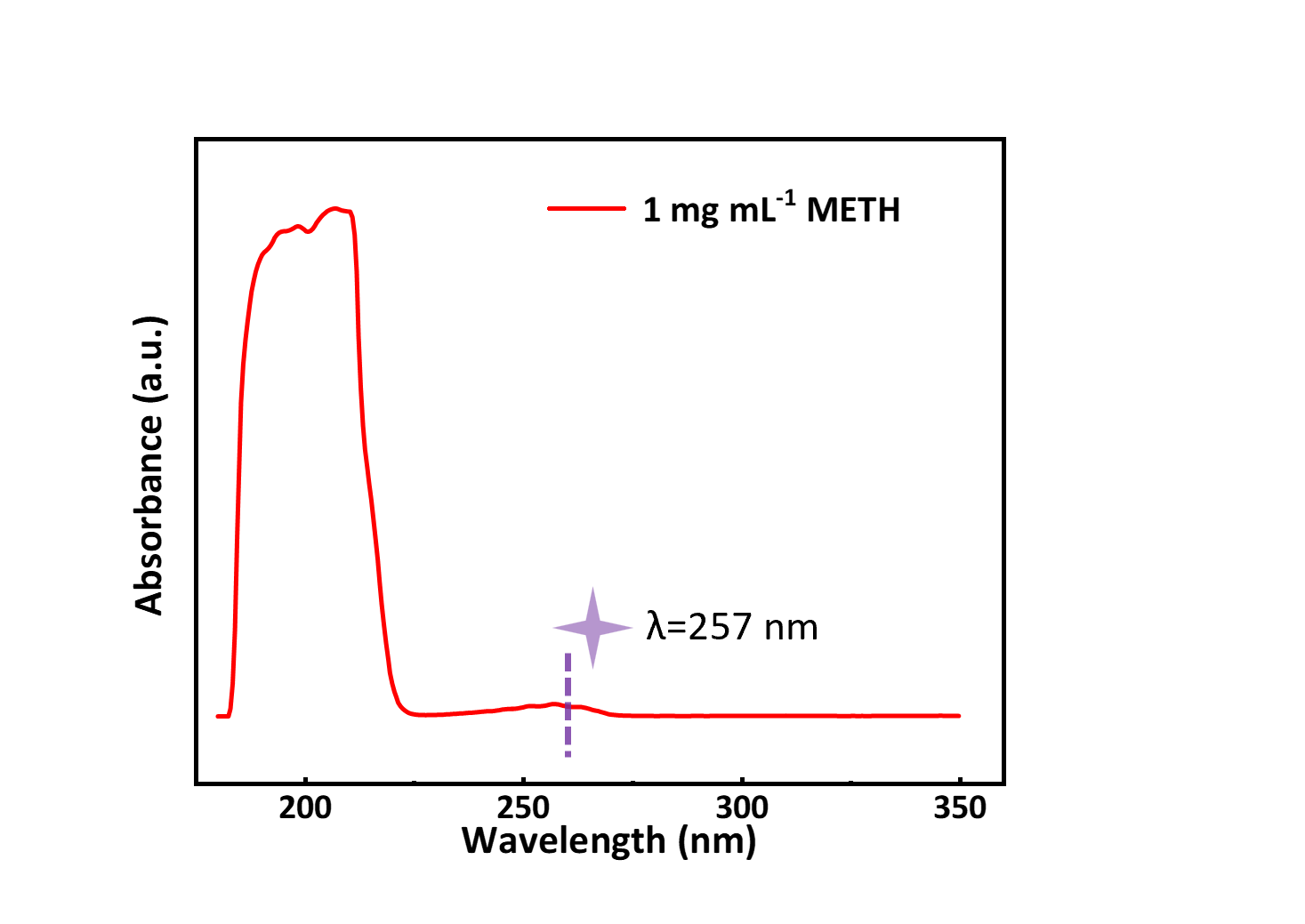


**Figure S5.** UV-vis absorption spectrum of a 1 mg mL^-1^ METH solution.


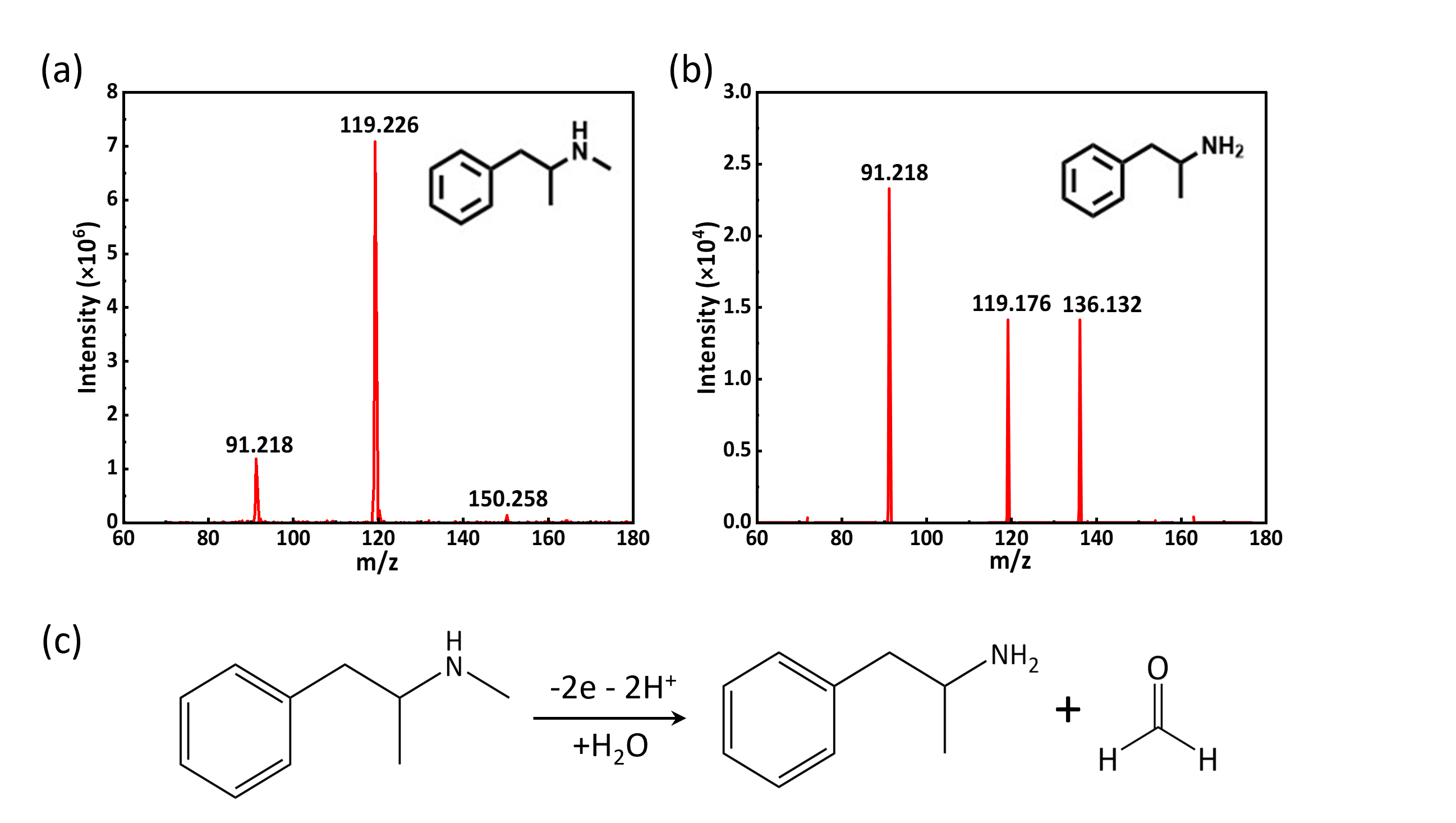


**Figure S6.** Mass spectrometry (MS) analysis. (a) MS spectrum of the METH standard. (b) MS spectrum of the solution after electrochemical oxidation, showing the reaction products. (c) The proposed mechanism for the electrochemical oxidation of METH.


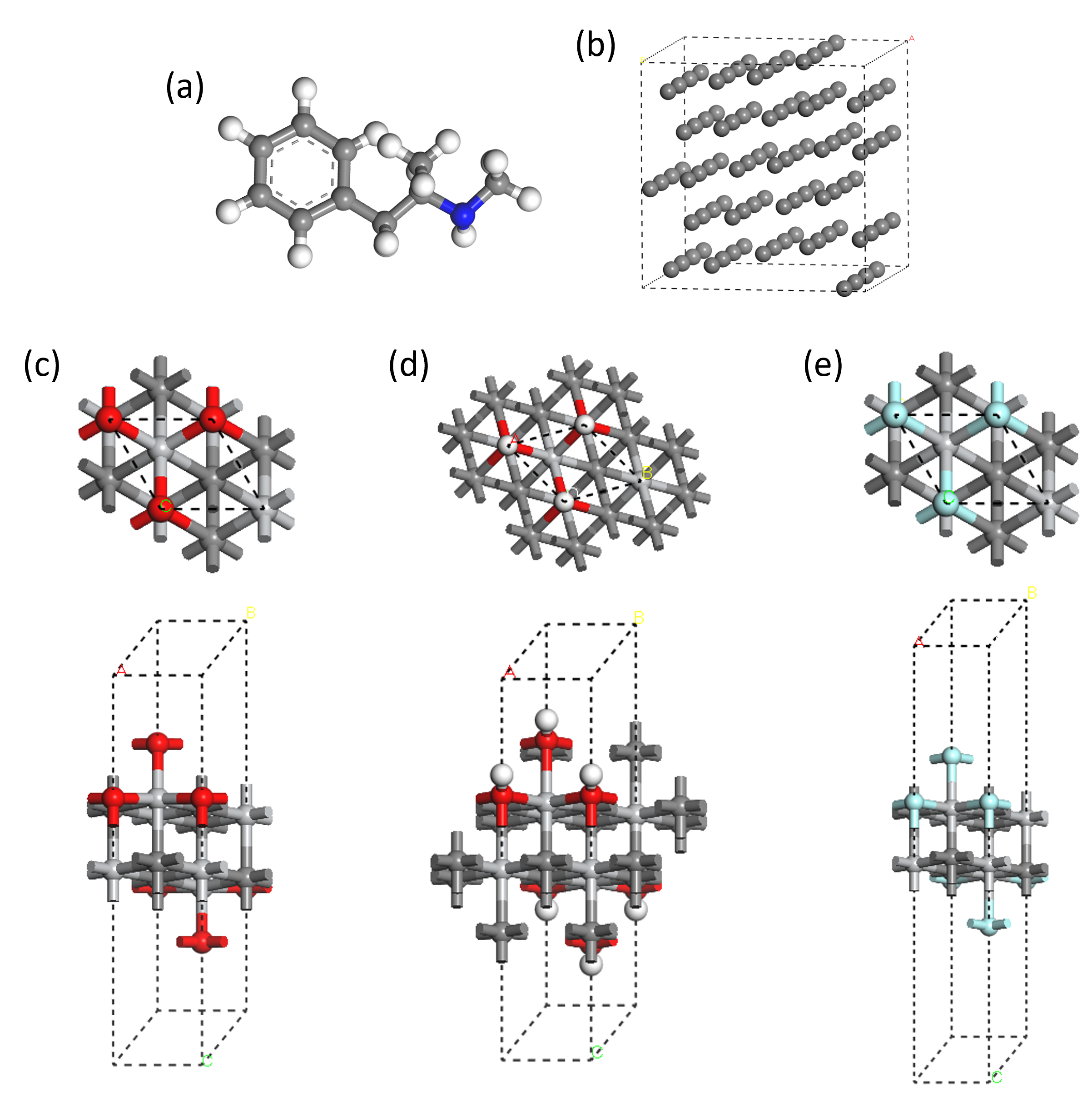


**Figure S7.** Structural models used for computational simulations. (a) METH molecule, (b) glassy carbon substrate, (c) Ti_3_C_2_O_2_, (d) Ti_3_C_2_(OH)_2_ and (e) Ti_3_C_2_F_2_ MXene surfaces. Titanium (Ti), carbon (C), oxygen (O), fluorine (F), hydrogen (H), and nitrogen (N) atoms are represented as grey, dark grey, red, blue, white, and dark bule, respectively.


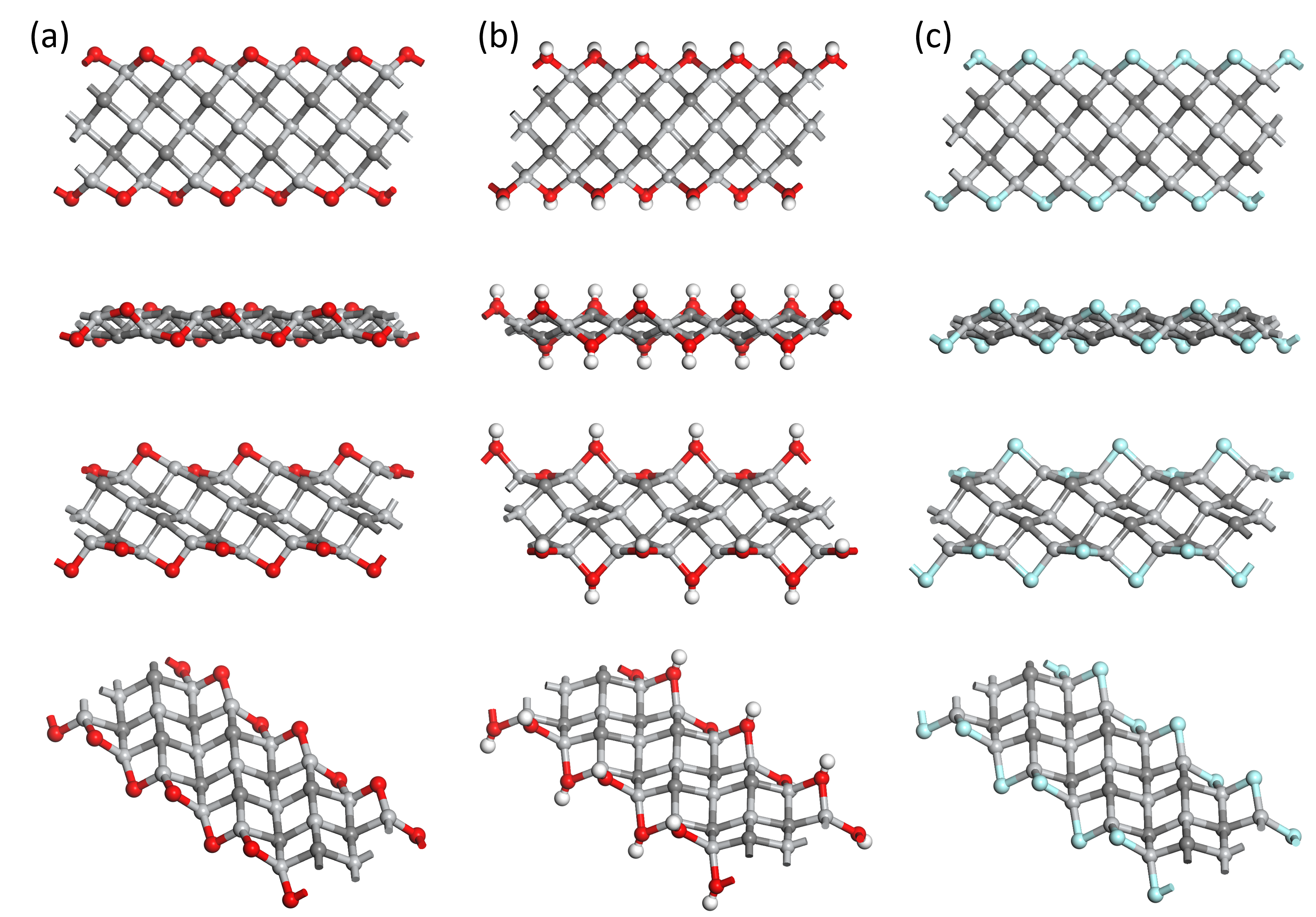


**Figure S8.** Optimized computational models of the MXene surfaces. (a) Ti_3_C_2_O_2_, (b) Ti_3_C_2_(OH)_2_ and (c) Ti_3_C_2_F_2_. Titanium (Ti), carbon (C), oxygen (O), fluorine (F), and hydrogen (H) atoms are represented as grey, dark grey, red, blue, and white, respectively.


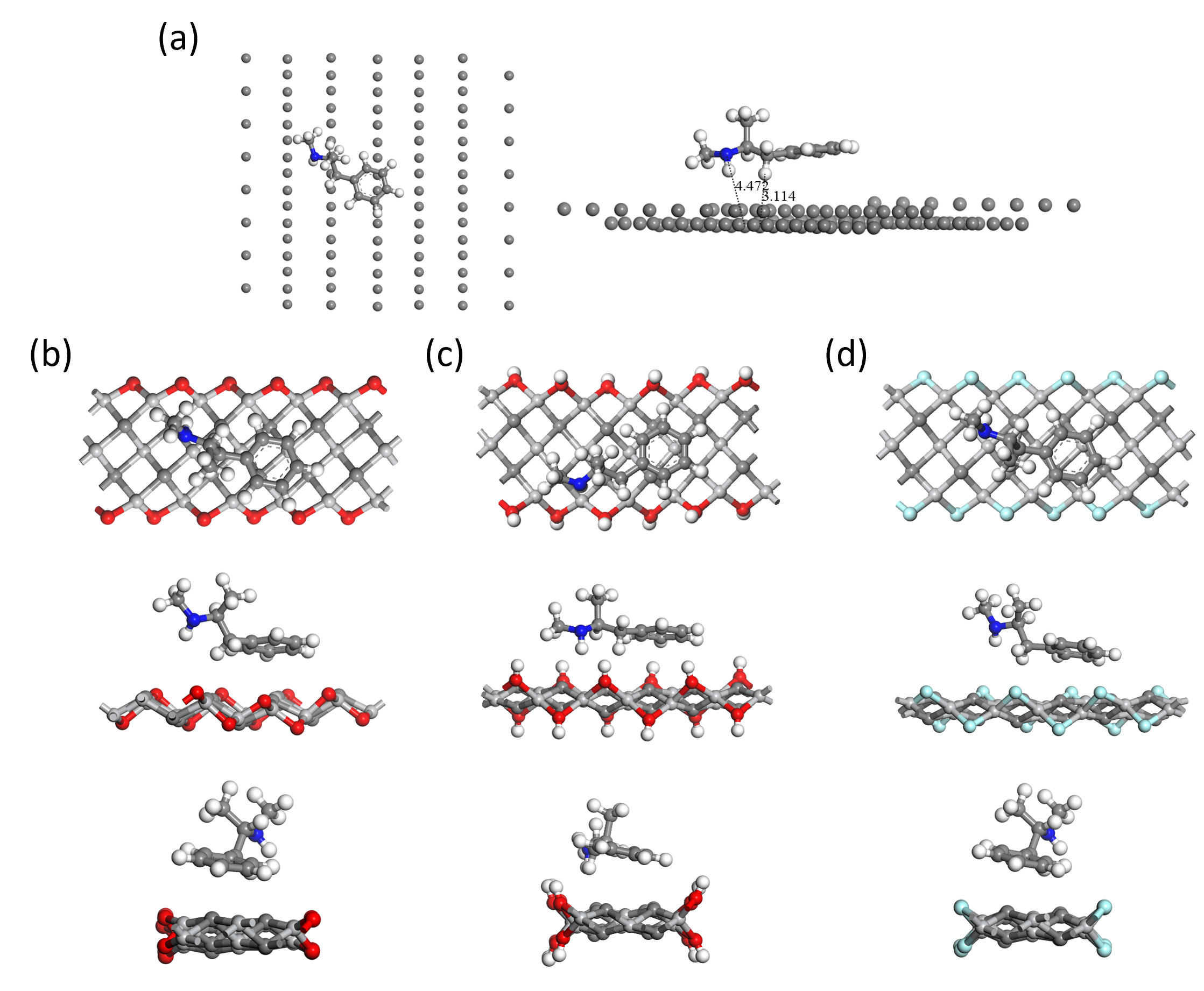


**Figure S9.** Final simulated configurations from simulations showing METH molecules interacting with (a) a glassy carbon surface, (b) Ti_3_C_2_O_2_, (c) Ti_3_C_2_(OH)_2_, and (d) Ti_3_C_2_F_2_ MXene surfaces. Titanium (Ti), carbon (C), oxygen (O), fluorine (F), hydrogen (H), and nitrogen (N) atoms are represented as grey, dark grey, red, blue, white, and dark bule, respectively.


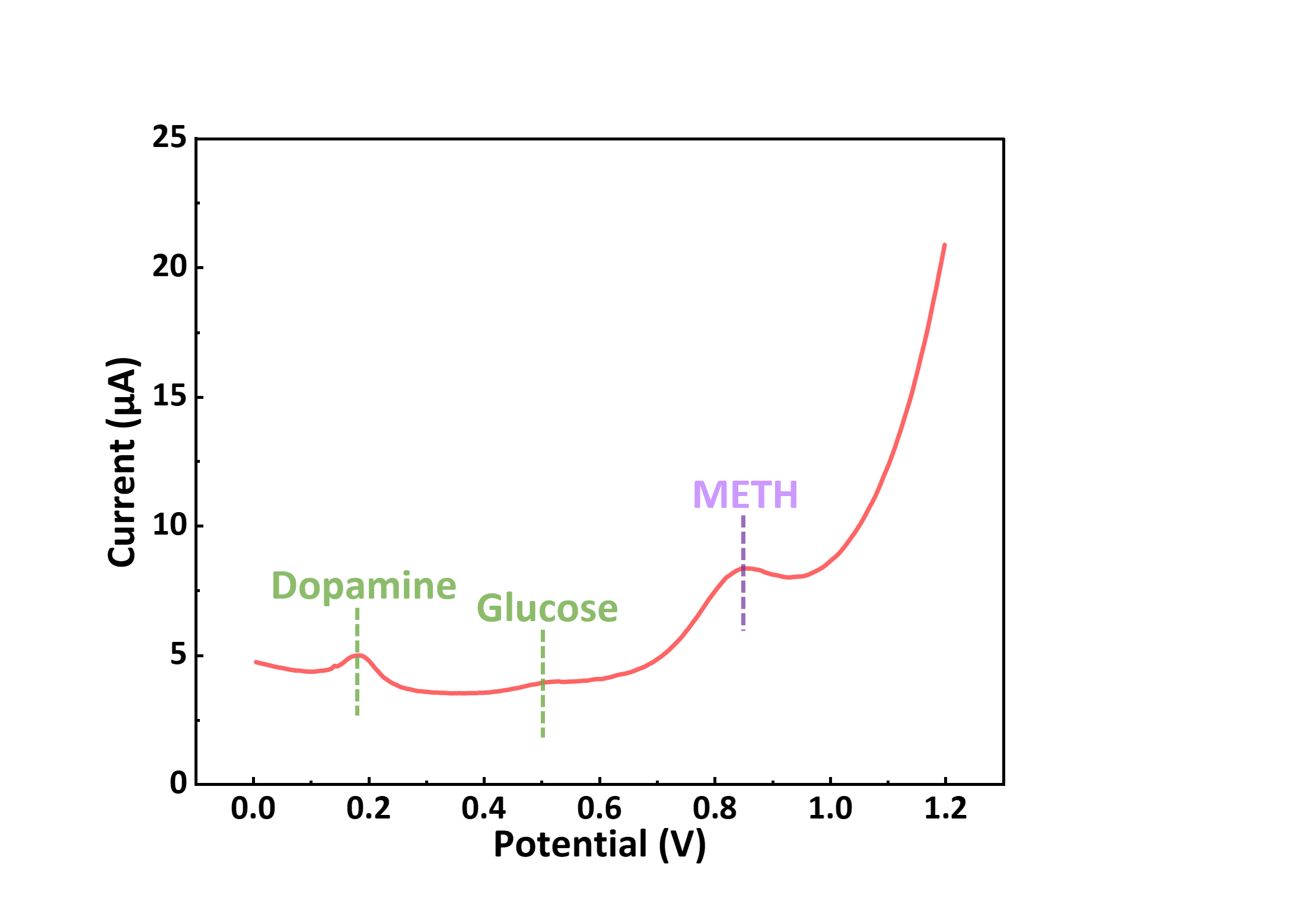


**Figure S10.** DPV response of the MX@Nf-modified sensor in rat serum, showing the distinct oxidation peak for METH and signals from endogenous interferents.

**Table S1.** XPS fitting results for MXene@Nafion modified GCE.

| Region | Binding energy (eV) | Assigned to |
| --- | --- | --- |
| Ti 2p_3/2_ (2p_1/2_) | 455.37 (461.45) | Ti-C |
|  | 456.52 (463.12) | Ti(II) |
|  | 459.56 (465.56) | Ti(IV) |
| O 1s | 530.91 | C-Ti-O_x_ |
|  | 532.41 | C-Ti-(OH)_x_ |
|  | 533.54 | S=O |
|  | 535.59 | S-O |
| C 1s | 282.29 | C-Ti-T_x_ |
|  | 284.80 | C-C |
|  | 286.30 | C-O |
|  | 289.24 | C=O |
|  | 290.76 | CF |
|  | 291.98 | CF_2_ |
|  | 293.34 | CF_3_ |
| F 1s | 685.85 | C-Ti-F_x_ |
|  | 689.11 | CF_2_ |
| S 2p_3/2_ (2p_1/2_) | 169.30 (170.48) | Sulfonate |

**Table S2.** Consistency results of the MX@Nf-modified GCE (*n* = 3).

| C_METH_ (μg mL^-1^) | 0.5 | 1 | 5 |
| --- | --- | --- | --- |
| RSD | 2.73% | 4.05% | 2.15% |

**Table S3.** Distances of METH with different surfaces.

|  | Ti_3_C_2_O_2_-METH | Ti_3_C_2_(OH)_2_-METH | Ti_3_C_2_F_2_-METH | GC-METH |
| --- | --- | --- | --- | --- |
| H-C | 2.617 Å | 2.705 Å | 2.551 Å | 3.114 Å |
| H-O | 3.136 Å | 2.924 Å | / | / |
| H-H | / | 2.531 Å | / | / |
| H-F | / | / | 3.160 Å | / |
| N-O | 5.059 Å | 3.926 Å | / | / |
| N-H | / | 3.440 Å | / | / |
| N-F | / | / | 4.994 Å | / |
| N-C | / | / | / | 4.472 Å |
